# Algorithmic Prognostication in Female Oncofertility Counseling: Ethical Challenges of Bias, Autonomy, and Predictive Uncertainty

**DOI:** 10.3390/healthcare14162538

**Published:** 2026-08-13

**Authors:** Huei-Ying Chiu, Ya-Ting Chuang, Simona Zaami, Tao-An Chen

**Affiliations:** 1 Department of Obstetrics and Gynecology, Show Chwan Memorial Hospital, Changhua 50009, Taiwan; 2 Surgical Intensive Care Unit, Department of Nursing, Show Chwan Memorial Hospital, Changhua 50009, Taiwan; 3 Department of Anatomical, Histological, Forensic and Orthopedic Sciences, Sapienza University of Rome, 00161 Rome, Italy; 4 Division of Respiratory Therapy, Department of Chest Medicine, Show Chwan Memorial Hospital, Changhua 50009, Taiwan; 5 Institute of Emergency and Critical Care Medicine, National Yang Ming Chiao Tung University, Taipei 112304, Taiwan

**Keywords:** fertility preservation, oncofertility, artificial intelligence, machine learning, predictive modeling, reproductive autonomy, shared decision-making, algorithmic bias, predictive uncertainty, medical ethics

## Abstract

**Highlights:**

**What are the main findings?**

- Algorithmic prognostication may improve individualized fertility-risk assessment and support timely fertility-preservation counseling for women undergoing cancer treatment.
- Predictive uncertainty, algorithmic bias, limited validation, and data heterogeneity present important ethical challenges that may affect reproductive equity and patient autonomy.

**What are the implications of the main findings?**

- Predictive algorithms should be implemented as decision-support tools rather than determinants of reproductive outcomes, with clear communication of uncertainty and model limitations.
- Responsible use of AI in oncofertility requires transparency, fairness evaluation, ongoing validation, human oversight, and regulatory safeguards to support informed and value-concordant decision-making.

**Abstract:**

Advances in machine learning, predictive analytics, and clinical prediction modeling have accelerated the development of algorithmic tools for estimating reproductive outcomes after cancer treatment. In female oncofertility counseling, these models may support individualized assessment of treatment-related amenorrhea, premature ovarian insufficiency, and fertility risk, thereby improving risk communication and timely fertility-preservation referral. However, their use raises ethical concerns beyond predictive accuracy. This narrative review examines algorithmic prognostication in female oncofertility counseling, focusing on predictive uncertainty, surrogate reproductive endpoints, missing data, heterogeneous datasets, limited external validation, algorithmic bias, reproductive inequity, and the influence of algorithmic authority on patient autonomy and shared decision-making. We argue that predictive algorithms should be understood as decision-support tools rather than determinants of reproductive futures. Responsible implementation requires transparency, explainability, fairness assessment, ongoing validation, and meaningful human oversight. Algorithmic risk estimates should be communicated as conditional and contextual probabilities within patient-centered counseling, ensuring that predictive tools support informed, transparent, and value-concordant fertility-preservation decisions for women facing cancer treatment.

## 1. Introduction

Fertility preservation (FP) has become an increasingly important domain of reproductive medicine, with cryopreservation of gametes, embryos, or gonadal tissue used to preserve reproductive potential before it is compromised by disease, medical treatment, or age-related decline [1,2,3]. Initially developed for patients exposed to gonadotoxic cancer therapies, FP technologies are now applied across broader clinical and non-clinical settings, including endometriosis, transgender care, and elective FP [1,2,4,5,6]. The expansion of FP practices has intensified ethical discussion surrounding reproductive autonomy, distributive justice, access inequality, commercialization, and the medicalization of reproductive risk [7,8,9,10].

FP is increasingly recognized not merely as a biomedical intervention, but as a socially and ethically mediated process shaped by cultural values, regulatory frameworks, and evolving concepts of reproductive choice [7,10,11,12]. Advances in cancer diagnosis and treatment have substantially improved survival among adolescents and young adults, increasing attention toward long-term survivorship and quality-of-life outcomes, including reproductive health and FP [3,13,14]. Breast cancer remains one of the most common malignancies affecting women worldwide and accounts for nearly one-quarter of all female cancers globally [13,15,16,17]. As the population of young cancer survivors continues to grow, fertility-related outcomes have become an increasingly important component of survivorship care [18,19]. Treatment-related infertility is now recognized as a major long-term complication among female cancer survivors and may affect 40–80% of women exposed to gonadotoxic therapies [16]. Chemotherapy-induced ovarian injury may lead to treatment-related amenorrhea (TRA), diminished ovarian reserve, premature ovarian insufficiency (POI), or early menopause [20,21,22]. The risk of ovarian dysfunction varies according to patient age, chemotherapy regimen, baseline ovarian reserve, and cumulative gonadotoxic exposure [4,23]. Radiotherapy involving pelvic, abdominal, or craniospinal fields may similarly expose the ovaries to ionizing radiation and accelerate follicular depletion [24,25,26,27]. Radiation-related gonadotoxicity is influenced by total dose, fractionation schedule, and age at treatment, with younger women generally demonstrating greater ovarian reserve resilience [24,27].

The reproductive consequences of cancer therapy extend beyond biological infertility. Approximately 70% of young women with early-stage breast cancer report future fertility intentions, while infertility-related concerns frequently contribute to psychological distress, decisional conflict, impaired quality of life, and long-term emotional burden [13,28,29,30]. Fertility concerns may also influence oncologic decision-making, including adherence to endocrine therapy and acceptance of recommended treatment regimens [29,30]. FP counseling has therefore become an increasingly important component of contemporary oncofertility care [16,18,31]. This transition has further increased demand for individualized reproductive risk assessment and clinical decision-support tools [18,32].

Recent advances in machine learning (ML), predictive analytics, and large-scale clinical data integration have accelerated the development of algorithmic prognostic tools designed to estimate reproductive outcomes after cancer treatment [13,33]. Compared with conventional regression-based prediction systems, ML approaches can incorporate complex nonlinear relationships, high-dimensional variables, and heterogeneous datasets without relying on strict prior assumptions [34,35,36]. Despite their clinical promise, algorithmic prognostic systems introduce important methodological and ethical challenges. Many predictive models are derived from heterogeneous retrospective datasets characterized by variable outcome definitions, extensive missing data, and limited external validation, all of which may compromise predictive accuracy, reliability, and generalizability [37,38]. These methodological limitations become ethical concerns when they contribute to inaccurate clinical recommendations, inequitable access to fertility-preservation counseling, or undue influence on patient autonomy and shared decision-making. Furthermore, algorithmic outputs may exert disproportionate influence over patients facing emotionally distressing and time-sensitive fertility decisions shortly after cancer diagnosis [39,40]. Risk estimates presented as objective numerical probabilities may shape reproductive choices, alter perceptions of future parenthood, or reinforce clinician-driven recommendations in ways that challenge reproductive autonomy and shared decision-making [37]. Beyond technical accuracy, the ethical implementation of predictive algorithms in oncofertility counseling therefore requires careful consideration of transparency, explainability, uncertainty communication, fairness, accountability, and patient-centered care [18,37,41]. While algorithmic prognostication may improve individualized counseling and facilitate timely FP discussions, poorly validated or insufficiently contextualized models may also contribute to decisional coercion, reproductive inequity, or misplaced confidence in predictive outputs [37,41,42].

This narrative review critically examines the ethical implications of algorithmic prognostication in female oncofertility counseling. Rather than providing a comparative evaluation of predictive model performance or a comprehensive review of regulatory policies, this review focuses on the ethical challenges arising from the clinical implementation of current predictive models. Drawing on recent predictive models for TRA, ovarian insufficiency, and fertility risk estimation, this review focuses on predictive uncertainty, data heterogeneity, missing-data handling, limited external validation, algorithmic bias, potential reproductive inequity, and the influence of algorithmic authority on patient autonomy and shared decision-making. By integrating technical limitations with ethical analysis, we aim to identify the conditions under which predictive algorithms may responsibly support, rather than replace, individualized fertility-related cancer care.

## 2. Materials and Methods

### 2.1. Review Design and Framework

This narrative review was conducted following Ferrari’s narrative review framework, which provides guidance for structuring, synthesizing, and reporting literature reviews in a narrative style [43]. The framework was used to guide a focused synthesis of the literature on algorithmic prognostication in female oncofertility counseling. The review was organized around five domains: predictive model development, reproductive risk estimation, predictive uncertainty, algorithmic bias and equity, and ethical implications for autonomy and shared decision-making.

### 2.2. Eligibility Criteria

Peer-reviewed English-language studies were eligible for inclusion if they examined female patients with cancer, young women with cancer, or female cancer survivors in relation to oncofertility, fertility preservation, fertility counseling, reproductive risk estimation, ovarian reserve, amenorrhea, premature ovarian insufficiency, infertility, or gonadotoxicity. Studies were also considered eligible if they addressed prognostic models, prediction models, risk calculators, algorithms, machine learning, artificial intelligence, or clinical decision-support tools relevant to reproductive outcomes after cancer treatment. Because this review focused on ethical implications, studies addressing bias, fairness, equity, autonomy, informed consent, transparency, explainability, uncertainty, risk communication, or shared decision-making were also included.

Studies were excluded if they did not focus on female reproductive outcomes, were unrelated to cancer or gonadotoxic treatment, did not address fertility risk prediction or algorithmic prognostication, or focused exclusively on non-oncology infertility prediction. Conference abstracts, editorials, and non-English publications were excluded.

### 2.3. Search Strategy

A structured search was conducted in PubMed and Ovid MEDLINE from database inception to May 2026. The PubMed search combined terms related to oncofertility, female cancer populations, reproductive outcomes, prediction models, artificial intelligence, machine learning, and ethical issues. Search terms included “oncofertility,” “fertility preservation,” “fertility counseling,” “reproductive counseling,” “female,” “women,” “young women,” “female cancer patients,” “ovarian reserve,” “anti-Müllerian hormone,” “AMH,” “antral follicle count,” “gonadotoxicity,” “amenorrhea,” “premature ovarian insufficiency,” “infertility,” “prediction model,” “prognostic model,” “risk prediction,” “risk calculator,” “algorithm,” “machine learning,” “artificial intelligence,” “ethics,” “bias,” “fairness,” “equity,” “autonomy,” “informed consent,” “transparency,” “explainability,” “uncertainty,” and “shared decision making.” For Ovid MEDLINE, controlled vocabulary and free-text terms were combined using Boolean operators. The search included Medical Subject Headings and keyword terms for neoplasms, female populations, fertility preservation, reproductive medicine, infertility, ovarian reserve, premature ovarian failure, prognosis, algorithms, artificial intelligence, machine learning, risk assessment, clinical decision support, medical ethics, bioethical issues, shared decision-making, health equity, and uncertainty. Search strategies were adapted according to the syntax of each database. Detailed search strategies for each database are provided in Supplementary Material File, with the PubMed search strategy presented in Supplementary File S1A and the Ovid MEDLINE search strategy presented in Supplementary File S1B.

In addition to database searches, the grey literature was searched using Google Scholar to supplement the identification of relevant conceptual, ethical, methodological, and implementation-related sources. Google Scholar searches used combinations of terms related to oncofertility, fertility preservation, female cancer survivors, prediction models, artificial intelligence, machine learning, algorithmic bias, reproductive autonomy, uncertainty, explainability, equity, and shared decision-making. The first several pages of results were screened for relevance, with emphasis on reports, guidelines, policy documents, consensus statements, and scholarly articles not captured in PubMed or Ovid MEDLINE. Reference lists of relevant grey literature sources were also manually reviewed. The grey literature was included when it provided substantive information on ethical, clinical, or implementation issues relevant to algorithmic prognostication in female oncofertility counseling.

### 2.4. Study Selection

Titles and abstracts were screened to identify potentially relevant records. Full texts of eligible or potentially eligible articles were then reviewed. Studies were selected based on their relevance to algorithmic prognostication, reproductive risk estimation, fertility preservation counseling, and ethical implications in female oncofertility care. Because this was a narrative review rather than a systematic review or meta-analysis, formal risk-of-bias assessment and quantitative pooling were not performed.

### 2.5. Data Extraction

Data were extracted on bibliographic details, study design, population, cancer type, reproductive outcome of interest, prediction method or algorithmic approach, model inputs, outcome definitions, validation strategy, handling of missing data, reported limitations, and ethical considerations. Particular attention was given to predictive uncertainty, data heterogeneity, external validation, algorithmic bias, reproductive equity, transparency, explainability, patient autonomy, informed consent, and shared decision-making.

### 2.6. Data Synthesis

Owing to heterogeneity in study designs, populations, cancer types, treatment exposures, reproductive outcomes, prediction methods, and ethical frameworks, findings were synthesized narratively rather than statistically. The synthesis was organized around the role of algorithmic prognostication in female oncofertility counseling, the limits of reproductive risk estimation, validation gaps and predictive bias, reproductive inequity, and the ethical conditions under which predictive algorithms may responsibly support individualized fertility-related cancer care.

### 2.7. Narrative Synthesis and Ethical Framework

The narrative synthesis was primarily informed by five key studies describing the development, validation, or implementation of predictive models for oncofertility counseling [13,16,24,27,32]. These core studies were supplemented by approximately 40 additional peer-reviewed reviews, clinical guidelines, ethical analyses, and regulatory publications that provided the broader clinical, methodological, and ethical context for the discussion. The ethical analysis presented in this review was informed by the four principles—respect for autonomy, beneficence, non-maleficence, and justice—which served as the conceptual framework for evaluating the ethical implications of algorithmic prognostication in female oncofertility counseling.

## 3. Algorithmic Prognostication in Female Oncofertility Counseling

Algorithmic prognostication in female oncofertility counseling should be understood as part of a broader movement from descriptive risk classification toward individualized reproductive risk estimation [13,16]. In this context, predictive algorithms are not merely technical tools for calculating ovarian failure, but clinical instruments that translate heterogeneous reproductive, oncologic, hormonal, and treatment-related data into counseling-relevant probabilities [13,16].

Recent developments in artificial intelligence (AI), predictive analytics, and clinical prediction modeling provide the methodological basis for this transition. In reproductive medicine, AI-based approaches have been proposed as tools for integrating multidimensional clinical, embryologic, and biological information into personalized decision-support systems [33]. More broadly, machine-learning methods have become increasingly important in predictive medicine because they can identify complex patterns across high-dimensional biomedical datasets that may not be fully captured by conventional statistical approaches [34]. In oncology, however, the clinical translation of machine-learning prediction models requires rigorous attention to model development, validation, reporting quality, and applicability to real-world clinical settings [35]. At the same time, recent methodological discussions suggest that the central issue is not whether ML is inherently superior to logistic regression, but whether the underlying data are sufficiently complete, consistent, representative, and clinically meaningful to support reliable prediction [36].

Within oncofertility, these general AI principles have begun to translate into reproductive risk estimation models that aim to support individualized counseling before gonadotoxic treatment. Machine-learning approaches have been used to predict TRA in young women with breast cancer by integrating multicenter FoRECAST datasets and applying cross-imputation methods to address extensive missing data [13]. Similar efforts have produced fertility risk calculators that estimate individualized probabilities of chemotherapy-related POI, thereby shifting counseling from broad categorical risk groups toward continuous patient-specific probability estimates [16]. These models illustrate how algorithmic prognostication may incorporate age, baseline ovarian reserve, hormonal biomarkers, treatment intensity, radiation exposure, prior therapy, and other patient-specific variables into clinically usable counseling tools [13,16].

Clinically, such tools may provide value by helping clinicians identify patients at higher reproductive risk earlier in the treatment pathway, prioritize timely referral to fertility-preservation specialists, and support more individualized discussions before gonadotoxic therapy begins [4,13,16,18]. When used as decision-support rather than decision-making instruments, algorithmic estimates may strengthen shared decision-making by making reproductive risks more explicit, comparable, and discussable within the limited time available after cancer diagnosis [13,16,18].

The non-AI and foundational literature further strengthen the clinical basis for algorithmic prognostication. Mathematical models of radiation-associated ovarian insufficiency demonstrate that age and ovarian radiation dose can be used to estimate reproductive lifespan reduction after cancer therapy [24]. Biological studies of chemotherapy-induced ovarian damage and ovarian reserve decline provide the mechanistic rationale for incorporating age, AMH, Follicle-stimulating hormone (FSH), treatment regimen, and cumulative gonadotoxic exposure into prediction models [20,21]. Current FP guidelines also emphasize early counseling before gonadotoxic treatment, which makes individualized risk estimation clinically meaningful rather than merely computational [4,18].

**Figure 1:**
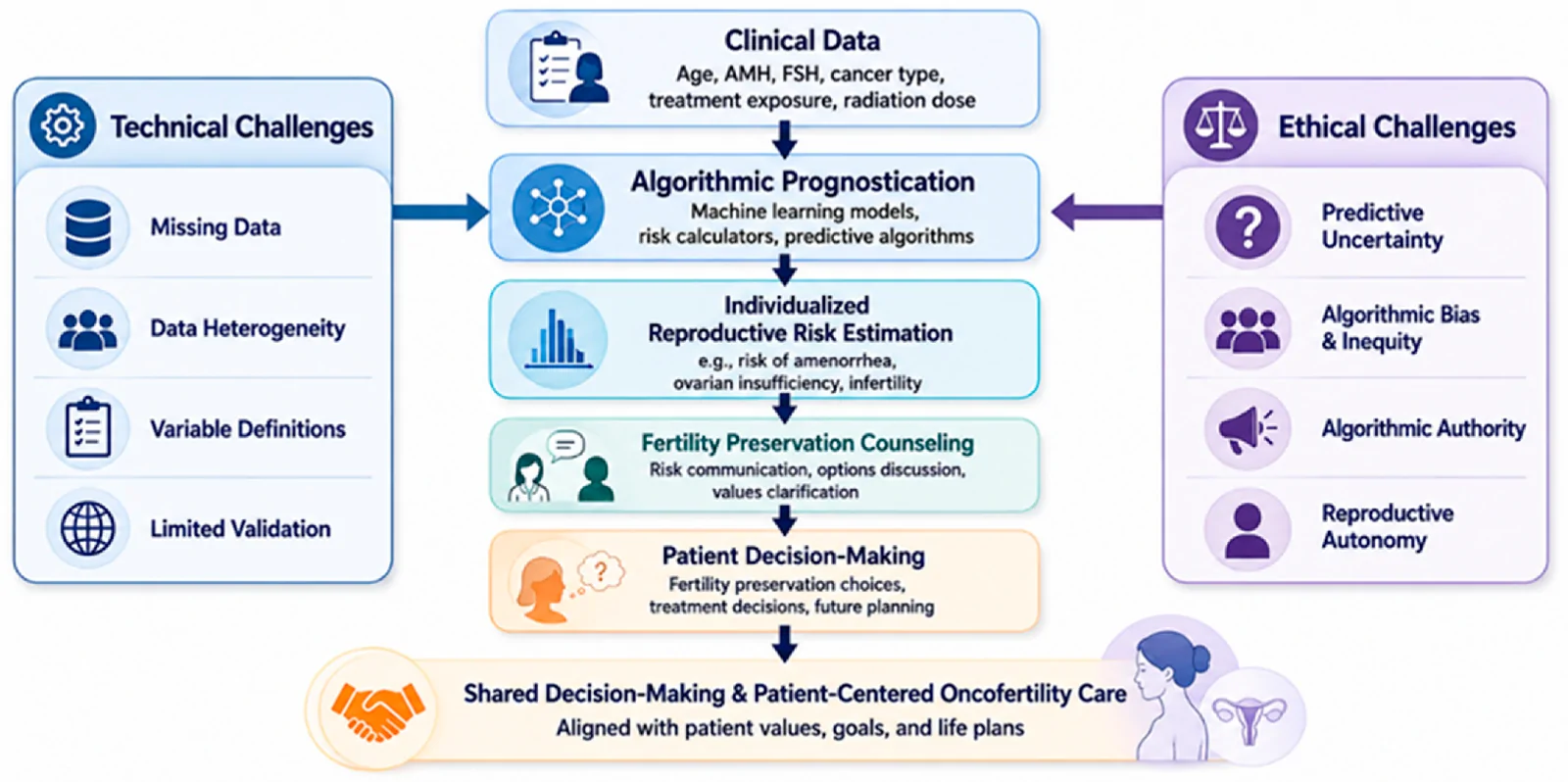
Conceptual Framework of Algorithmic Prognostication in Female Oncofertility Counseling.

Therefore, algorithmic prognostication in female oncofertility counseling is best viewed as a layered clinical-ethical practice: reproductive AI provides the technical possibility of personalization; predictive medicine provides the methodological framework; the oncology prediction literature defines standards for validation and reporting; the data-quality literature clarifies the limits of model performance; and oncofertility models translate these principles into counseling for young women facing gonadotoxic therapy [13,16,33,34,35,36]. This framing avoids treating algorithms as autonomous decision-makers and instead positions them as decision-support tools whose value depends on transparent interpretation, clinically valid data, and careful integration into shared reproductive decision-making [13,35,36]. The relationship between algorithmic prediction, clinical counseling, and the technical and ethical factors that shape reproductive risk estimation is summarized in Figure 1.

## 4. Predictive Uncertainty and the Limits of Reproductive Risk Estimation

In this review, technical limitations are distinguished from their ethical implications. Methodological limitations may reduce predictive performance or contribute to unequal model performance, whereas ethical concerns arise when these limitations influence clinical decisions, compromise patient autonomy, or result in inequitable care.

Despite the increasing sophistication of algorithmic prognostic tools, reproductive risk estimation after cancer treatment remains intrinsically uncertain because ovarian function is not a fixed endpoint but a dynamic biological process shaped by age, endocrine recovery, baseline ovarian reserve, cumulative gonadotoxic exposure, and long-term follicular depletion [13,16,21]. The biological rationale for this uncertainty is supported by evidence that chemotherapy and radiation may impair ovarian reserve through different mechanisms and that reproductive lifespan varies according to age, treatment exposure, and residual follicular reserve [20,22,25].

A major source of uncertainty arises from the use of surrogate reproductive outcomes. Current models frequently estimate TRA, ovarian insufficiency, or biochemical ovarian failure rather than directly predicting future pregnancy, live birth, or long-term reproductive capacity [13,16,21,32]. Amenorrhea after chemotherapy may be clinically meaningful, but it does not necessarily indicate irreversible infertility because some women may later recover menstruation, whereas others may experience delayed ovarian decline despite temporary recovery [13]. Similarly, diminished ovarian reserve does not always correspond to immediate infertility, particularly among younger women who retain residual reproductive potential [16]. Predictive uncertainty is also amplified by heterogeneity across datasets, populations, treatment protocols, and outcome definitions. Multicenter reproductive prediction models often combine data collected from different countries, institutions, clinical trials, and observational cohorts, each with distinct follow-up schedules, treatment exposures, hormonal measurements, and definitions of ovarian failure [13,16,32]. Even when models address similar clinical questions, differences in amenorrhea definition, timing of outcome assessment, inclusion criteria, and endpoint construction may influence model discrimination, calibration, and comparability [13,16,44,45,46,47,48,49].

Missing data represent another important boundary of algorithmic reproductive prediction. In multicenter datasets, missingness may arise from incomplete clinical records, invalid values, inconsistent variable collection, or feature mismatch across contributing studies [13,50,51,52]. Cross-imputation using K-nearest neighbor algorithms may allow more patients and variables to be retained for model development, but imputed values remain estimated rather than directly observed [53,54,55,56]. Similarly, multiple imputation using chained equations relies on assumptions about the missing-data mechanism, which may not fully reflect the biological or clinical processes generating missingness [56,57,58]. External validation further illustrates the limits of reproductive risk estimation. A model may perform well in internal validation but show weaker generalizability when applied to independent cohorts with different patient characteristics, treatment patterns, outcome prevalence, or data completeness [13,16,35]. These limitations suggest that prediction accuracy depends not only on algorithmic method, but also on dataset quality, measurement consistency, representativeness, and clinical context [35,36,56]. Radiation-based models reveal a parallel limitation: although ovarian dose and treatment age can be used to estimate ovarian insufficiency or reproductive lifespan reduction, such models may not fully capture combined chemotherapy exposure, asymmetric ovarian dose, uterine radiation effects, or long-term patient-centered reproductive outcomes [24,25,26].

Current predictive tools are designed to estimate different reproductive outcomes rather than to function as directly competing prediction models. Consequently, their predictive performance cannot be directly compared. Instead, they exhibit distinct methodological limitations—including differences in statistical approaches, sample size, outcome definitions, data heterogeneity, and the extent of external validation—which contribute to varying degrees of predictive uncertainty. These methodological differences should be recognized during clinical counseling because uncertainty in oncofertility prognostication is not only statistical but also has important ethical implications. Fertility is a future-oriented outcome shaped by ovarian biology, survivorship trajectory, partnership status, reproductive intentions, socioeconomic resources, access to fertility preservation, and evolving personal values [4,11,18]. Accordingly, numerical risk estimates should be communicated as conditional approximations rather than deterministic forecasts. The responsible use of prognostic algorithms therefore requires transparent communication of surrogate endpoints, incomplete data, imputation assumptions, limitations in external validation, and the residual uncertainty surrounding individualized predictions [13,35,36,37].

## 5. Predictive Bias, Validation Gaps, and Reproductive Inequity

The performance of predictive algorithms depends not only on computational methodology but also on the quality, representativeness, and completeness of the data used for model development [34,36,59,60]. Consequently, algorithmic predictions may inherit biases embedded within historical clinical datasets, institutional practices, and healthcare systems [37,38,60]. Rather than eliminating subjectivity, predictive models may inadvertently encode and reproduce existing disparities present in the populations from which they are derived [37,38].

A major source of predictive bias arises from limited population representativeness. Many reproductive prediction models have been developed using retrospective datasets derived primarily from specialized academic centers, clinical trial populations, or patients with complete follow-up data [13,32,35]. Patients who are older, socioeconomically disadvantaged, treated in resource-limited settings, or lost to follow-up may therefore be underrepresented during model development [35,38]. As a result, algorithm performance observed in development cohorts may not be fully generalizable to broader patient populations. Validation gaps further contribute to uncertainty regarding fairness and reliability. Although internal validation may demonstrate favorable discrimination metrics, such findings do not necessarily guarantee performance across different institutions, geographic regions, treatment protocols, or demographic groups [16,35]. In reproductive prediction research, external validation cohorts are frequently smaller than development cohorts and may contain substantial amounts of imputed data [13]. Consequently, reported predictive performance may overestimate real-world clinical utility when algorithms are applied to populations that differ from the original training environment [35,36,50,61].

As discussed above, missing data represent not only a technical limitation of reproductive risk estimation but also a potential source of predictive bias. In multicenter reproductive datasets, incomplete clinical records, invalid values, inconsistent variable collection, and feature mismatch across contributing studies may require cross-imputation or multiple imputation before model development [13,50,51,52]. However, when missingness is unevenly distributed across institutions, treatment settings, or patient subgroups, imputed values may reproduce the structure of the better-documented populations rather than accurately representing under-documented groups [13,16,35,56,57,58]. Consequently, the same missing-data problem that limits predictive certainty may also contribute to algorithmic bias, particularly if patients with poorer access to follow-up, fertility counseling, hormonal testing, or specialist referral are less completely represented in the training data.

The challenge of reproductive inequity extends beyond technical validation. Access to FP services remains uneven across healthcare systems and socioeconomic groups [4,18,62]. Financial barriers, geographic disparities, insurance coverage limitations, cultural factors, and differences in specialist referral patterns may substantially influence who receives fertility counseling and fertility-preservation interventions [4,11,18,35,38,62]. Consequently, prediction models developed primarily in populations with relatively good access to fertility services may not accurately reflect reproductive decision-making among underserved groups.

Importantly, predictive bias may influence clinical recommendations even when model performance appears acceptable. Risk estimates generated by algorithms may affect the timing of fertility-preservation referral, clinician perception of reproductive prognosis, and patient willingness to pursue fertility-preservation procedures [13,16]. If prediction errors disproportionately affect specific patient groups, seemingly objective algorithms may contribute to unequal allocation of reproductive opportunities [37,38].

These concerns have prompted growing calls for fairness-oriented model evaluation. Contemporary AI ethics frameworks increasingly recommend assessing predictive performance across demographic, socioeconomic, and clinical subgroups rather than relying solely on overall discrimination metrics [37,38]. Similarly, oncology prediction-model guidelines emphasize transparent reporting, external validation, calibration assessment, and evaluation of clinical applicability before implementation [35,37,38].

Taken together, predictive bias, incomplete validation, and reproductive inequity highlight the limitations of viewing algorithmic outputs as universally applicable or inherently objective. Responsible implementation therefore requires not only technical accuracy but also ongoing assessment of representativeness, fairness, transparency, and equitable access across diverse reproductive populations.

## 6. Reproductive Autonomy, Algorithmic Authority, and Shared Decision-Making

In female oncofertility counseling, predictive algorithms may influence decisions that extend beyond clinical risk estimation to future parenthood, identity, family planning, and life goals [4,18]. Because fertility-preservation decisions are often made soon after cancer diagnosis, patients may encounter algorithmic risk estimates during a period of emotional distress, time pressure, and uncertainty [11,40]. This creates the risk of algorithmic authority. Numerical predictions may appear objective, neutral, or definitive, even when they are shaped by surrogate outcomes, missing data, model assumptions, and validation limits [37,38]. In reproductive counseling, a high-risk estimate may increase pressure to pursue FP, whereas a low-risk estimate may reduce perceived urgency despite residual uncertainty [13,16].

The ethical concern is not only whether the prediction is accurate, but also how it shapes the patient’s understanding of possible futures [11]. Fertility decisions involve value-sensitive tradeoffs, including cancer-treatment priorities, treatment delay, financial burden, future relationship status, cultural expectations, and personal meanings of parenthood [4,11]. Therefore, algorithmic outputs should not be presented as recommendations, but as conditional information to support deliberation [13,16]. Shared decision-making remains essential in this context. Clinicians should explain what the model predicts, what it does not predict, which variables influence the estimate, and how applicable the result is to the individual patient [35,37]. This includes clarifying that predicted amenorrhea, ovarian insufficiency, or fertility risk does not necessarily determine future parenthood [13,16].

Algorithmic risk estimates may also shape how patients imagine their future reproductive lives [11,13,16,37,38]. A high-risk prediction may make biological parenthood appear urgent, fragile, or nearly impossible without immediate intervention, whereas a low-risk prediction may lead patients to underestimate future reproductive vulnerability [13,16]. In this way, algorithmic prognostication does not merely inform a pre-existing choice; it may influence how patients construct expectations about future parenthood, weigh uncertainty, and define what reproductive possibility means after cancer [11,37,38]. Respect for reproductive autonomy therefore requires that predictive outputs be framed as one source of information within a broader conversation about values, hopes, fears, and acceptable uncertainty, rather than as authoritative judgments about whether parenthood is likely or worth pursuing [4,11,18,37].

**Figure 2:**
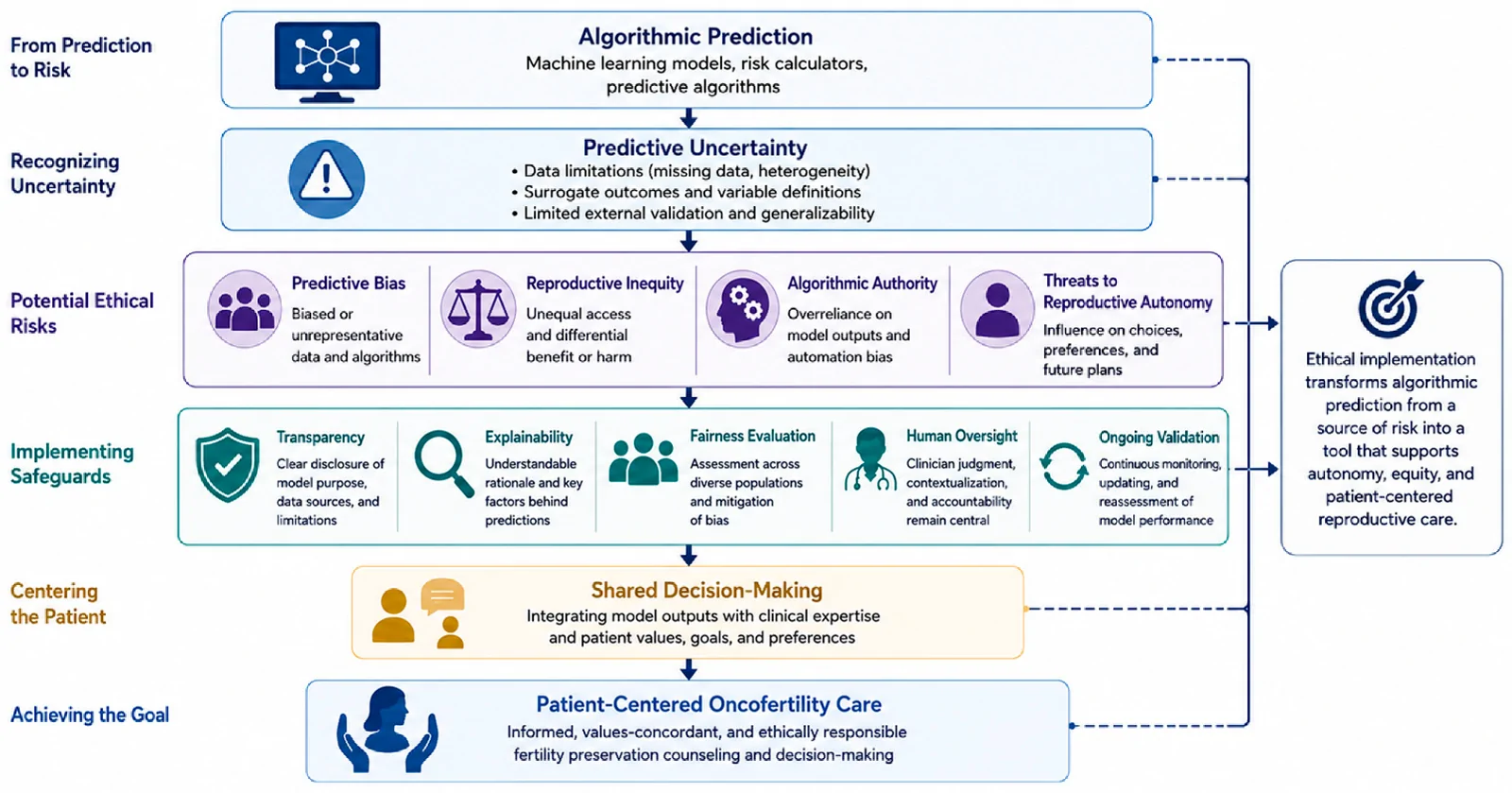
Ethical Pathway for Responsible Implementation of Algorithmic Prognostication.

In this context, human interpretation, patient values, and clinician–patient dialogue should remain central to algorithm-assisted counseling [18,37]. The goal is not to allow algorithms to decide reproductive futures, but to use them carefully to support informed, transparent, and value-concordant fertility-preservation decisions. The ethical safeguards required for the responsible implementation of algorithmic prognostication in female oncofertility counseling are summarized in Figure 2.

## 7. Ethical Requirements for Responsible Implementation

The ethical challenges discussed above do not imply that predictive algorithms should be excluded from oncofertility counseling. Rather, they highlight the need for safeguards that ensure algorithmic tools are implemented in ways that support, rather than undermine, patient-centered care [35,37]. Responsible implementation therefore requires attention not only to predictive performance but also to transparency, explainability, fairness, accountability, and respect for patient autonomy [37,38,63].

Transparency represents a foundational requirement. Clinicians and patients should be informed about the outcome being predicted, the variables included in the model, and the limitations associated with missing data, surrogate endpoints, and external validation [13,35,64]. Without adequate transparency, numerical predictions may be interpreted as definitive facts rather than probabilistic estimates derived from specific datasets and assumptions [37,50,65].

Closely related to transparency is explainability. Patients may reasonably wish to understand why a particular prediction was generated and which factors contributed most strongly to their estimated reproductive risk [37,38,66,67]. Explainability is particularly important in oncofertility because reproductive decisions are highly personal and often involve time-sensitive interventions with significant long-term implications, such as oocyte cryopreservation, embryo cryopreservation, and ovarian tissue preservation [1,4,18].

Fairness and equity should also be considered essential components of responsible implementation. Prediction models should be evaluated across diverse patient populations to identify potential performance differences related to age, socioeconomic status, healthcare access, ethnicity, or treatment setting [37,38,63,64]. Reliance solely on overall performance metrics may obscure clinically important disparities affecting underrepresented groups [35,36,68].

Human oversight remains equally important. Predictive algorithms may assist fertility counseling, but they cannot replace clinical judgment, individualized assessment, or patient preferences [13,37]. Clinicians remain responsible for contextualizing risk estimates, communicating uncertainty, and ensuring that decisions align with the patient’s values and goals [1,4,18,39,63].

Finally, ongoing validation and post-implementation evaluation are necessary because model performance may change when algorithms are applied to new populations, healthcare systems, or treatment environments [13,35,63,64]. Responsible implementation should therefore be viewed as a continuous process rather than a one-time demonstration of predictive accuracy [37,38,63,64].

Taken together, the ethical use of algorithmic prognostication in female oncofertility counseling requires more than accurate prediction. It requires transparent communication, explainable models, equitable evaluation, meaningful human oversight, and ongoing validation to ensure that predictive tools support informed and value-concordant reproductive decision-making [1,4,13,18,35,37,39,63,64,68].

## 8. Regulatory Safeguards and European Governance

Algorithmic prognostication in female oncofertility counseling holds great promise for enhancing personalized care, but must be approached with careful ethical, legal and regulatory consideration [12,13,37,38,63]. The European scenario shows that addressing bias requires rigorous data evaluation and algorithmic fairness measures. Protecting patient autonomy demands transparency, clear communication, and shared decision-making frameworks [69,70]. Managing predictive uncertainty involves honest dialogue about the limits of AI predictions and their implications for patient care [37,38,63]. This section extends the preceding ethical analysis by showing how European regulatory frameworks operationalize the ethical requirements of fairness, autonomy, transparency, and accountability in clinical AI governance.

In Europe, the ethical challenges of bias, autonomy, and predictive uncertainty in algorithmic prognostication for female oncofertility counseling are regulated through a combination of legal frameworks, ethical guidelines, and regulatory oversight focused on AI, healthcare, and data protection. From the perspective of algorithmic bias, the European Union (EU) Artificial Intelligence Act (AI Act) is especially relevant because it transforms fairness and representativeness from abstract ethical ideals into regulatory obligations for high-risk healthcare AI systems. The AI Act, which came into force on 1 August 2024, classifies AI systems used in healthcare as high-risk and imposes strict requirements for transparency, bias mitigation, and accuracy [69,70]. The Act mandates representative datasets, bias testing, and transparency, including a right to explanation, to reduce discriminatory bias and support patient autonomy and informed consent [69,70]. The biggest risks of bias in AI for oncofertility stem from data bias, where AI models are trained on historical clinical data that may not represent all patient populations [4,11,18,35,38,62,71]. If the training data lacks diversity in race, ethnicity, socioeconomic status, age, or cancer types, the AI may produce less accurate or unfair predictions for underrepresented groups, leading to disparities in fertility counseling and treatment options [34,36,59,60,72]. Measurement bias arises from inaccurate or inconsistent data collection methods, such as variability in fertility markers or cancer staging, which can affect the reliability of AI prognostication and misinform patients and clinicians [4,18,62,73]. AI systems may perpetuate existing clinical biases when trained on historical decisions shaped by subjective judgments, while disparities in technological, economic, and healthcare resources can limit access to AI tools, thereby exacerbating inequities in fertility preservation counseling and care [74].

The General Data Protection Regulation (GDPR), on the other hand, is designed to govern another key element: the processing of personal health data used in AI systems, ensuring lawful, fair, and transparent data use [75]. From the perspective of reproductive autonomy, GDPR is important because fertility prediction depends on highly sensitive personal and reproductive health data [4,11,18,37]. It protects patient privacy and upholds autonomy by mandating informed consent for data processing, which is critical in fertility prediction tools [76]. GDPR also supports the right to explanation regarding automated decisions affecting individuals. Relevant studies on GDPR and AI provide further insights into this relationship.

The Medical Device Regulation (MDR) applies to AI software used in clinical settings, including fertility counseling, regulating safety, efficacy, and risk management [77]. From the perspective of predictive uncertainty, MDR is relevant because clinical AI software requires validation, risk management, and ongoing monitoring after implementation [37,38,63]. MDR requires clinical validation and ongoing monitoring of AI tools to ensure reliability and manage predictive uncertainty. Guidance on AI and medical devices is available through official EU health documents and resources on how ever-evolving innovation navigates EU medical device and AI rules.

AI is increasingly used to improve infertility diagnosis and assisted reproductive technology outcomes by predicting pregnancy success and optimizing treatment protocols [4,18,78]. Ethical frameworks emphasize the importance of counseling that integrates AI predictions as decision-support tools, preserving patient autonomy and addressing predictive uncertainty [4,11,18,37,79]. Examples and discussions on harnessing AI for cancer care in Europe and ethical dilemmas in oncofertility highlight the importance of patient-centered counseling alongside AI use.

Thus, these European frameworks should not be viewed merely as external legal requirements, but as practical mechanisms for embedding ethical safeguards into algorithm-assisted oncofertility counseling. European regulations like the AI Act, GDPR, and MDR create a comprehensive framework to meet the need to regulate bias, protect patient autonomy, and manage predictive uncertainty in AI-driven female oncofertility counseling [69,70,75,77]. These laws ensure AI tools are transparent, fair, and used ethically within clinical decision-making, supported by ongoing clinical oversight and patient-centered counseling.

## 9. Limitations

Several limitations should be acknowledged. First, this narrative review focuses primarily on the ethical implications of algorithmic prognostication in female oncofertility counseling and does not provide a systematic assessment of predictive performance across all available fertility prediction models. Consequently, the discussion emphasizes conceptual and ethical concerns rather than quantitative comparisons of model accuracy, calibration, or clinical utility.

Second, the current evidence base remains limited by the relatively early stage of algorithm development within oncofertility. Most available studies focus on TRA, ovarian insufficiency, or fertility-risk estimation, whereas evidence regarding the impact of predictive algorithms on real-world counseling outcomes, fertility-preservation uptake, decisional conflict, patient satisfaction, and long-term reproductive outcomes remains sparse [14,17].

Third, many ethical concerns discussed in this review are prospective rather than empirically established. Although theoretical risks related to algorithmic bias, automation bias, reproductive inequity, and algorithmic authority are supported by the broader healthcare AI literature, direct evidence evaluating these phenomena in oncofertility counseling remains limited [39,40,69,70].

Finally, the rapid evolution of AI, machine-learning methodologies, and fertility-preservation practices may influence the relevance of current ethical challenges over time. Future research should address several important knowledge gaps, including prospective multicenter validation of predictive models across diverse populations, standardized outcome definitions, transparent model reporting and explainability, assessment of algorithmic fairness and bias, and evaluation of the real-world impact of predictive tools on shared decision-making, fertility-preservation uptake, patient satisfaction, and long-term reproductive outcomes. Addressing these knowledge gaps through prospective, multidisciplinary research will be essential to strengthen the evidence base supporting the ethical and clinical implementation of AI-assisted oncofertility counseling, while further validating and refining the ethical framework proposed in this review [31,37,39,69,70].

## 10. Conclusions

Algorithmic prognostication has the potential to enhance female oncofertility counseling by supporting individualized fertility-risk estimation and shared decision-making. However, current evidence remains limited by methodological heterogeneity, incomplete external validation, and the early stage of clinical implementation. While concerns regarding algorithmic bias, automation bias, reproductive inequity, and reduced patient autonomy are supported conceptually and by the broader healthcare AI literature, direct empirical evidence in oncofertility remains limited. Therefore, predictive algorithms should be implemented as decision-support tools that complement, rather than replace, clinical judgment. Moving forward, clinicians should communicate predictive uncertainty transparently, researchers should prioritize prospective validation, model explainability, and fairness across diverse populations, and regulators should establish governance frameworks that ensure transparency, accountability, and equitable implementation. Such multidisciplinary efforts will be essential to achieve the responsible integration of AI into patient-centered oncofertility care.

## Data Availability

Data sharing is not applicable to this article because no datasets were generated or analyzed during the current study. All information discussed in this review was obtained from publicly available published sources.

## Supplementary Materials

The following supporting information can be downloaded at: https://www.mdpi.com/article/10.3390/healthcare14162538/s1, Supplementary Material S1: PubMed Search Strategy (File S1A); Ovid MEDLINE Search Strategy (File S1B).

## Abbreviations

The following abbreviations are used in this manuscript:

AI	Artificial intelligence
AI Act	Artificial Intelligence Act
AMH	Anti-Müllerian hormone
EU	European Union
FP	Fertility preservation
FSH	Follicle-stimulating hormone
GDPR	General Data Protection Regulation
MDR	Medical Device Regulation
ML	Machine learning
POI	Premature ovarian insufficiency
TRA	Treatment-related amenorrhea
