# Supplementary material for: Algorithmic Prognostication in Female Oncofertility Counseling: Ethical Challenges of Bias, Autonomy, and Predictive Uncertainty
Source: Healthcare (Basel). 2026 Aug 13;14(16):2538. doi: 10.3390/healthcare14162538 (PMC13512539; doi:10.3390/healthcare14162538)
Supplement: Supplementary file 1 [file healthcare-14-02538-s001.zip › healthcare-4411863-supplementary.pdf]

# Supplementary Material S1. Detailed Search Strategies

Search strategies were adapted according to the indexing system, controlled vocabulary, and search syntax of each database. Detailed search strategies for PubMed and Ovid MEDLINE are provided below.

## Supplementary Material S1A. PubMed Search Strategy

Three complementary PubMed search strategies were used to identify the literature related to female oncofertility, reproductive risk estimation, predictive modeling, artificial intelligence, machine learning, and ethical issues.

### PubMed Search 1: Oncofertility, prediction models, and ethics

(
"oncofertility"[Title/Abstract]
OR "fertility preservation"[Title/Abstract]
OR "fertility counseling"[Title/Abstract]
OR "reproductive counseling"[Title/Abstract]
)
AND
(
female[Title/Abstract]
OR women[Title/Abstract]
OR woman[Title/Abstract]
OR "young women"[Title/Abstract]
OR "female cancer patient*"[Title/Abstract]
OR "female cancer survivor*"[Title/Abstract]
)
AND
(
cancer[Title/Abstract]
OR oncology[Title/Abstract]
OR neoplasm*[Title/Abstract]
OR chemotherapy[Title/Abstract]
OR radiotherapy[Title/Abstract]
)
AND
(
"prognostic model*"[Title/Abstract]

OR "prediction model*"[Title/Abstract]
OR "risk prediction"[Title/Abstract]
OR "risk calculator*"[Title/Abstract]
OR "clinical prediction model*"[Title/Abstract]
OR algorithm*[Title/Abstract]
OR "machine learning"[Title/Abstract]
OR "artificial intelligence"[Title/Abstract]
)
AND
(
ethic*[Title/Abstract]
OR bias[Title/Abstract]
OR fairness[Title/Abstract]
OR equity[Title/Abstract]
OR autonomy[Title/Abstract]
OR "reproductive autonomy"[Title/Abstract]
OR "informed consent"[Title/Abstract]
OR transparency[Title/Abstract]
OR explainability[Title/Abstract]
OR uncertainty[Title/Abstract]
OR "shared decision making"[Title/Abstract]
)

**PubMed Search 2: Oncofertility, ovarian reserve, and prediction models**

(
  "oncofertility"[Title/Abstract]
  OR "fertility preservation"[Title/Abstract]
)
AND
(
  female[Title/Abstract]
  OR women[Title/Abstract]
  OR "young women"[Title/Abstract]
  OR "female cancer patient*"[Title/Abstract]
)
AND
(
  "ovarian reserve"[Title/Abstract]
  OR "anti-Mullerian hormone"[Title/Abstract]

OR "anti-Müllerian hormone"[Title/Abstract]
OR AMH[Title/Abstract]
OR "antral follicle count"[Title/Abstract]
OR AFC[Title/Abstract]
OR gonadotoxicity[Title/Abstract]
OR amenorrhea[Title/Abstract]
OR "chemotherapy-induced amenorrhea"[Title/Abstract]
OR infertility[Title/Abstract]
OR "premature ovarian insufficiency"[Title/Abstract]
OR "premature menopause"[Title/Abstract]
)
AND
(
"prediction model*"[Title/Abstract]
OR "prognostic model*"[Title/Abstract]
OR "risk prediction"[Title/Abstract]
OR "risk calculator*"[Title/Abstract]
OR algorithm*[Title/Abstract]
OR "machine learning"[Title/Abstract]
)
AND
(
cancer[Title/Abstract]
OR oncology[Title/Abstract]
OR chemotherapy[Title/Abstract]
OR radiotherapy[Title/Abstract]
)

**PubMed Search 3: Female cancer populations, reproductive outcomes, and predictive modeling**

(
women[Title/Abstract]
OR female[Title/Abstract]
OR "young women"[Title/Abstract]
)
AND
(
cancer[Title/Abstract]
OR oncology[Title/Abstract]

OR chemotherapy[Title/Abstract]
OR radiotherapy[Title/Abstract]
)
AND
(
"ovarian reserve"[Title/Abstract]
OR "anti-Mullerian hormone"[Title/Abstract]
OR "anti-Müllerian hormone"[Title/Abstract]
OR AMH[Title/Abstract]
OR gonadotoxicity[Title/Abstract]
OR amenorrhea[Title/Abstract]
OR "premature ovarian insufficiency"[Title/Abstract]
OR infertility[Title/Abstract]
)
AND
(
"prediction model*"[Title/Abstract]
OR "risk prediction"[Title/Abstract]
OR "prognostic model*"[Title/Abstract]
OR "risk calculator*"[Title/Abstract]
OR algorithm*[Title/Abstract]
OR "machine learning"[Title/Abstract]
)

## Supplementary Material S1B. Ovid MEDLINE Search Strategy

The Ovid MEDLINE search combined controlled vocabulary and free-text terms for cancer, female populations, fertility preservation, ovarian reserve, predictive modeling, artificial intelligence, machine learning, and ethical issues.

1. exp Neoplasms/
2. (cancer* or neoplasm* or tumor* or tumour* or malignan* or oncology).ti,ab,kf.
3. 1 or 2
4. exp Female/
5. (female* or woman or women or girl* or adolescent girl* or young women or reproductive-age women).ti,ab,kf.
6. 4 or 5
7. 3 and 6
8. exp Fertility Preservation/

9. exp Reproductive Medicine/
10. exp Infertility, Female/
11. exp Ovarian Reserve/
12. exp Premature Ovarian Failure/
13. (oncofertility or "fertility preservation" or "fertility counselling" or "fertility counseling" or "reproductive counselling" or "reproductive counseling").ti,ab,kf.
14. ("oocyte cryopreservation" or "embryo cryopreservation" or "ovarian tissue cryopreservation" or "ovarian suppression").ti,ab,kf.
15. ("ovarian reserve" or "anti-Mullerian hormone" or "anti-Müllerian hormone" or AMH or "antral follicle count" or AFC).ti,ab,kf.
16. (gonadotoxic* or amenorrhea or "chemotherapy-induced amenorrhea" or "premature ovarian insufficiency" or "premature ovarian failure" or "premature menopause" or infertility).ti,ab,kf.
17. 8 or 9 or 10 or 11 or 12 or 13 or 14 or 15 or 16
18. exp Prognosis/
19. exp Algorithms/
20. exp Artificial Intelligence/
21. exp Machine Learning/
22. exp Risk Assessment/
23. exp Decision Support Systems, Clinical/
24. ("prognostic model*" or "prediction model*" or "predictive model*" or "clinical prediction model*" or "risk prediction" or "risk calculator*" or "risk score*" or algorithm*).ti,ab,kf.
25. ("artificial intelligence" or AI or "machine learning" or "deep learning" or "neural network*" or "clinical decision support").ti,ab,kf.
27. exp Ethics, Medical/
28. exp Bioethical Issues/
29. exp Decision Making, Shared/
30. exp Health Equity/
31. exp Uncertainty/
32. (ethic* or bioethic* or bias or "algorithmic bias" or fairness or equity or justice).ti,ab,kf.
33. (autonomy or "reproductive autonomy" or "patient autonomy" or "informed consent").ti,ab,kf.
34. (transparency or explainability or interpretability or accountability or responsibility).ti,ab,kf.
35. (uncertainty or "predictive uncertainty" or "risk communication" or "shared decision making" or "shared decision-making").ti,ab,kf.

36. 27 or 28 or 29 or 30 or 31 or 32 or 33 or 34 or 35 or 36 or 37
37.7 and 17 and 26 and 38
